# Prognostic impact of the time of admission and discharge from the intensive care unit

**DOI:** 10.5935/0103-507X.20170010

**Published:** 2017

**Authors:** Héctor Eduardo Moreira, Federico Verga, Marcelo Barbato, Gastón Burghi

**Affiliations:** 1 Terapia Intensiva, Hospital Maciel - Montevideo, Uruguay.

**Keywords:** Patient admission, Patient discharge, Prognosis, Mortality, Intensive care units

## Abstract

**Objective:**

To determine the impact of the day and time of admission and discharge from the intensive care unit on mortality.

**Methods:**

Prospective observational study that included patients admitted to the intensive care unit of the *Hospital Maciel* in Montevideo between April and November 2014.

**Results:**

We analyzed 325 patients with an average age of 55 (36 - 71) years and a SAPS II value of 43 (29 - 58) points. No differences were found in the mortality of patients in the intensive care unit when time of admission (35% on the weekend versus 31% on weekdays, p = ns) or the hour of entry (35% at night versus 31% in the daytime, p = ns) were compared. The time of discharge was associated with higher hospital mortality rates (57% for weekend discharges versus 14% for weekday discharges, p = 0.000). The factors independently associated with hospital mortality after discharge from the intensive care unit were age > 50 years (OR 2.4, 95%CI, 1.1 - 5.4) and weekend discharge (OR 7.7, 95%CI, 3.8-15.6).

**Conclusion:**

This study identified the time of discharge from the intensive care unit as a factor that was independently associated with hospital mortality.

## Abstract (es)

**Objetivo:**

Determinar el impacto en la mortalidad del día y horario de admisión y alta de la unidad de cuidados intensivos.

**Métodos:**

Estudio prospectivo observacional, que incluyó pacientes admitidos entre abril y noviembre de 2014 en la unidad de cuidados intensivos del Hospital Maciel de Montevideo.

**Resultados:**

Se analizaron 325 pacientes con una edad de 55 (36 - 71) años y un valor SAPS II de 43 (29 - 58) puntos. No se encontraron diferencias en la mortalidad en unidad de cuidados intensivos de los pacientes al comparar el momento de ingreso (35% el fin de semana *versus* 31% en los días laborales, p = ns) ni el horario de ingreso (nocturnos 35% *versus* diurnos 31%, p = ns). El momento de egreso se asoció a mayor mortalidad en el hospital (57% en egresos del fin de semana versus 14% en días laborales, p = 0,000). Los factores independientemente asociados a mortalidad hospitalaria luego del alta de unidad de cuidados intensivos fueron la edad mayor de 50 años (OR 2,4; IC95%, 1,1 - 5,4) y el egreso durante el fin de semana (OR 7,7; IC95%, 3,8 - 15,6).

**Conclusión:**

Este estudio logró identificar al momento del alta de la unidad de cuidados intensivos como un factor independientemente asociado a mortalidad hospitalaria.

## INTRODUCTION

Mortality in the intensive care unit (ICU) is closely linked to patients' clinical characteristics and severity. However, there are other factors, such as the time or day of admission to and discharge from the ICU, that impact the prognosis of critical patients.^(1,2)^

Bell et al.^(2)^ conducted a study in Canada that included the records of millions of patients and found that the day and time at which ICU admission occurs has an impact on patients' hospital survival. This timing is related to access to various technological resources for diagnosis.^(2-5)^

However, other studies did not find differences in hospital stay or mortality between patients admitted during the week and those admitted on the weekend, nor were they able to find differences according to the time of admission.^(6,7)^

Although the results remain controversial, the time of ICU admission has been extensively studied. Conversely, although the ICU discharge time may also have a special prognostic impact, this has been much less frequently evaluated in the literature. The deterioration in the quality and quantity of care that occurs when a patient is discharged may play a role in hospital mortality, and the transfer of patients to an area with a reduced capacity for care, as may occur in the hospital room, is among the transitions that carries the greatest risk for patient care.^(8)^ So-called "off-hour" discharges - those that occur on weekends or at night (after 19:00 or 20:00) - are associated with adverse patient outcomes, including increased readmission to the ICU and increased mortality.^(9-11)^ Laupland et al., in their study that included 7,380 patients, showed that discharges to the ward at the beginning of the weekend (Friday in the afternoon) were associated with an increased risk of dying in the hospital.^(12)^

The prognostic impact of the time of admission to or discharge from the ICU has not been studied in our setting. Evaluating this fact and identifying the factors associated with a worse prognosis are critical to strengthening health care systems and generating patient safety strategies that improve patient outcomes.

## METHODS

This report describes a prospective, non-interventional, observational cohort study. We analyzed a cohort of patients older than 18 years consecutively admitted between April 1 and November 1, 2014, to the multipurpose ICU of a tertiary public hospital with 250 beds.

This intensive care unit has 24 beds divided into 19 intensive care beds and 5 moderate care beds.

The work model followed in the ICU includes 4 intensive care physicians in charge of 6 patients 24 hours a day, every day of the week. Three additional staff doctors attend for 8 hours a day, participating in patient care in the mornings and attending clinical meetings in the afternoon. These meetings involve the doctors and nurses of the service and focus on making decisions related to the care and discharge of the patients on the unit. In the ICU, there is a university nurse for every 6 patients and a nursing assistant for every 2 patients.

In the internment floor, the medical staff performs their activities from 8:00 to 14:00 from Monday to Friday. Outside of those hours, the care of patients on that floor shifts to 2 on-call doctors who confer for patient evaluations as required by the nursing staff. On the floor, each nurse is in charge of 8 patients, and each nursing assistant cares for 8 to 12 patients.

Patients are considered discharged from the unit upon their admission to the internment floor. Discharges are determined at the UCI clinical meeting that takes place in the afternoon; consequently, most discharges occur in the afternoon. As a consequence, the room staff has no contact with the patient until the following morning.

All patients younger than 18 years were excluded, as were those admitted to preoperative elective surgery whose stay was presumed to be less than 48 hours. These patients were excluded due to their low severity scores and their relatively short stay in the ICU and in the hospital.^(13)^

Data were collected using a standardized questionnaire and a pre-designed checklist. The data collected from all patients included sociodemographic and hospitalization information at the time of admission (age, sex, source, type of admission, comorbidities, severity score - Simplified Acute Physiology Score II (SAPS II) - in the first 24 hours, time and day of admission to the ICU, diagnosis and treatments performed at admission); ICU discharge data (day of discharge, indication of limits for therapeutic effort, and status at discharge); and finally, hospital evolution.

The following operating variables were defined: weekdays were from Monday at 8 AM to Friday at 7:59 AM; weekends were from Friday at 8 AM until Monday at 7:59 AM.

Daytime admissions occurred between 8:00 AM and 7:59 PM. Night admissions occurred between 8 PM and 7:59 AM. Off-hours discharges occurred after 8:00 PM.

This study was approved by the Ethics Committee of the *Hospital Maciel*, and the included patients gave prior informed consent.

### Statistical analysis

The analysis was performed using the statistical package IBM SPSS version 21. The data are expressed as percentage or as median (quartiles 25% - 75%) for continuous variables. Categorical variables were compared using the chi square test or Fisher's exact test (with Yates correction when indicated). Continuous variables were compared with the Student *t* test or the rank-sum Mann-Whitney U test*,* according to the distribution of the variable. Statistical significance was set at p < 0.05. Variables associated with hospital mortality with p < 0.10 were included in a multivariate logistic regression model.

## RESULTS

**Figure 1:**
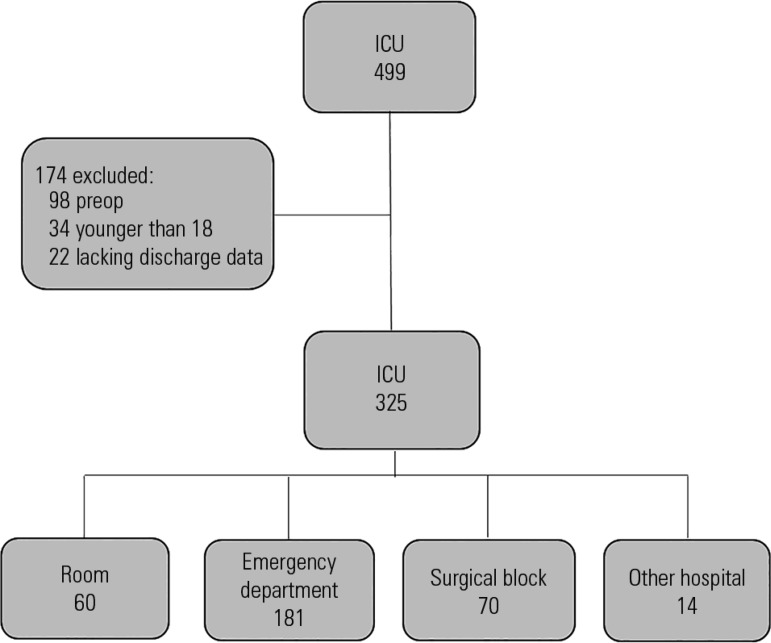
Structure for patient selection. ICU - intensive care unit.

During the study period, 499 admissions to the ICU were recorded. A total of 174 patients were excluded (Figure 1). The 325 patients included in the study were predominantly male (61.8%), with a median age of 55 years (36 - 71) and a SAPS II value of 43 (29 - 58). The average ICU stay was 9 (4 - 16) days. The main comorbidities were heart disease (28%) and chronic obstructive disease (23%). The ICU admissions came mainly from the emergency department (ED; n = 181; 56%). Half of the admissions (163) occurred at night (8:00 PM - 07:59 AM), and 39% (127) occurred during the weekend (Friday 8:00 AM to Monday 7:59 AM). We identified 23 (7%) patients who were readmitted to the ICU. The remaining characteristics are presented in table 1.

**Table 1:** Characteristics of the population and comparisons based on the day and time of admission

Characteristics	Total	Weekdays (N = 198)	Weekend (N = 127)	p value	Daytime admission (N = 163)	Nighttime admission (N = 162)	p value
N	325	198	127		163	162	
Age	55 (36 - 71)	52 (35 - 71)	59 (39 - 71)	ns	58 (38 - 72)	53 (32 - 70)	ns
Female	124 (38%)	72 (36)	52 (41)	ns	65 (40)	59 (36)	ns
SAPS II	43 (29 - 58)	42 (28 - 54)	46 (30 - 64)	ns	45 (32 - 56)	42 (26 - 61)	ns
Days before ICU admission	0 (0 - 1)	0 (0 - 1)	0 (0 - 1)	ns	0 (0 - 1)	0 (0 - 1)	ns
Days in ICU	9 (4 - 16)	8,5 (4 - 16)	10 (4 - 15)	ns	10 (4 - 17)	8 (4 - 15)	ns
Origin				ns			ns
Room	60 (19)	40 (20)	20 (16)		33 (20)	27 (17)	
Surgical block	70 (22)	43 (22)	27 (21)		32 (20)	38 (23)	
Emergency	181 (56)	105 (53)	76 (60)		95 (58)	86 (54)	
Another hospital	14 (4)	10 (5)	4 (3)		4 (2)	10 (6)	
IMV	270 (83)	163 (82)	107 (84)	ns	136 (83)	134 (83)	ns
Vasoactive drugs	232 (71)	139 (70)	93 (73)	ns	123 (75)	109 (67)	ns
RRT	12 (4)	8 (4)	8 (3)	ns	9 (5)	3 (2)	ns
Central venous access	321 (99)	196 (99)	125 (98)	ns	159 (97)	162 (100)	ns
Arterial access	262 (81)	158 (80)	104 (81)	ns	131 (80)	131 (81)	ns
Comorbidities				ns			ns
EPOC	75 (23)	48 (24)	27 (21%)		43 (26)	32 (20)	
Cardiopathy	90 (28)	55 (28)	35 (28)		50 (31)	40 (25)	
Cancer	16 (5)	11 (6)	5 (4)		7 (4)	9 (6)	
CKD	25 (8)	17 (8)	8 (6)		17 (10)	8 (5)	
HIV	10 (3)	5 (2)	5 (4)		6 (4)	4 (2)	
Hospital stay	20 (11 - 29)	19 (10 - 29)	21 (14 - 31)	ns	20 (12 - 29)	19,5 (10 - 30)	ns
Mortality in ICU	107 (33)	62 (31)	45 (35)	ns	51 (31)	56 (35)	ns

ns - non-significant; SAPS II - Simplified Acute Physiology Score II; ICU - intensive care unit; IMV - invasive mechanical ventilation; RRT - renal replacement therapy; COPD - chronic obstructive pulmonary disease; CKD - chronic kidney disease; HIV - human immunodeficiency virus; LTE - limitation of therapeutic effort. The results are expressed as number and percentage or median (25% - 75%).

There were no differences in the population characteristics according to the day or time of admission. Mortality did not differ according to the day or time of admission (31% weekday admission versus 35% weekend admission; p = ns; 31% daytime admission versus 35% nighttime admission; p = ns) (Table 1).

**Figure 2:**
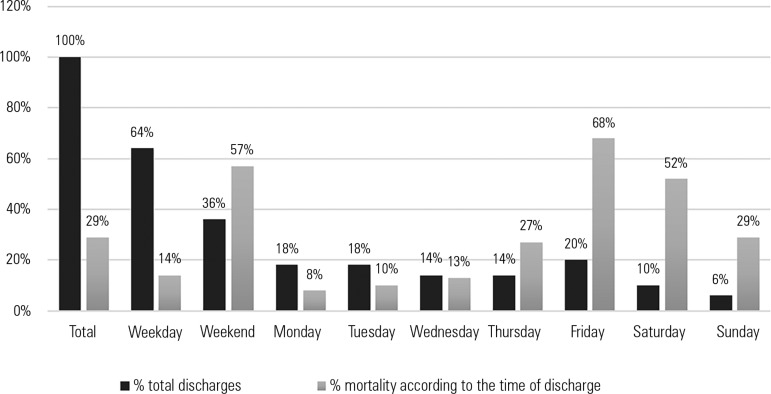
Mortality according to the time of discharge from the intensive care unit.

Of the patients discharged from the ICU (218), 30% (64) died in the hospital after ICU discharge. The ICU discharges were distributed as follows: 139 patients (64%) left the ICU on weekdays, and 79 (36%) left during the weekend (Figure 2). Patients discharged on the weekend had a significantly higher mortality (57% versus 14%; p = 0.000) (Table 2).

**Table 2:** Characteristics and mortality of patients based on the day of discharge from the intensive care unit

Characteristics	Weekday discharge (N = 138)	Weekend discharge (N = 79)	p value
Female	43 (31)	29 (37)	ns
SAPS II	37 (26 - 46)	36 (23 - 43)	ns
Days in ICU	10 (6 - 18)	10 (5 - 22)	ns
Origin			ns
Room	22 (16)	15 (19)	
Surgical block	33 (24)	20 (25)	
Emergency	78 (56)	41 (52)	
Another hospital	6 (4)	3 (4)	
IMV	103 (75)	61 (79)	ns
Vasoactive drugs	63 (46)	30 (39)	ns
RRT	8 (6)	6 (8)	ns
Central venous access	133 (97)	73 (95)	ns
Arterial access	81 (58)	42 (55)	ns
Comorbidities			ns
EPOC	25 (18)	23 (29)	
Cardiopathy	29 (21)	22 (28)	
Cancer	2 (1,5)	4 (5)	
CKD	7 (5)	4 (5)	
HIV	5 (3,5)	1 (1,5)	
Tracheostomy	33 (24)	27 (35)	ns
Hospital stay	21(12 - 29,5)	16 (9,75 - 29)	ns
LTE	7 (5)	5 (6)	ns
Died in the hospital	19 (14)	45 (57)	0,000

ns - non-significant; SAPS II - Simplified Acute Physiology Score II; ICU - intensive care unit; IMV - invasive mechanical ventilation; RRT - renal replacement therapy; COPD - chronic obstructive pulmonary disease; CKD - chronic kidney disease; HIV - human immunodeficiency virus; LTE - limitation of therapeutic effort. The results are expressed as percentage or median (25% - 75%).

Among the patients discharged from the ICU, factors associated with hospital mortality in the univariate analysis were SAPS II score > 35 (36% versus 21%; p = 0.017), weekend discharge (57% versus 14%, p = 0.000), the presence of chronic obstructive pulmonary disease (43% versus 26%, p = 0.031), history of heart disease (46% versus 25%, p = 0.005), and tracheostomy during the ICU stay (42% versus 25%, p = 0.019) (Table 3). Table 3 shows that the bivariable analysis found that age > 50 years was a factor associated with post-ICU mortality (43% versus 16%; p = 0.000).

**Table 3:** Bivariate analysis of factors associated with hospital mortality

Characteristics	Died in the hospital (with the characteristics)	Died in the hospital (without the characteristics)	p value
Age > 50 years	46/108 43	18/109 16	0.000
SAPS II > 35	43/118 36	21/99 21	0.017
Comorbidities			
CKD	4/11 36	60/206 29	0.7
EPOC	20/47 43	44/170 26	0.031
Cardiopathy	23/50 46	41/167 25	0.005
Cancer	4/6 67	60/211 28	0.06
HIV	2/6 33	62/211 29	1
MV	51/166 31	13/51 25	0.5
Vasopressors	27/92 29	37/121 31	0.8
Hemodialysis	7/14 50	57/198 29	0.13
Tracheostomy	25/59 42	39/154 25	0.019
Weekend discharge	45/79 57	19/138 14	0.000

SAPS II - Simplified Acute Physiology Score II; CKD - chronic kidney disease; COPD - chronic obstructive pulmonary disease; HIV - human immunodeficiency virus; MV - mechanical ventilation. The results are expressed as specific value, total and percentage.

Using multivariate logistic regression analysis, the factors associated with hospital mortality after discharge from the ICU were discharge during the weekend (odds ratio [OR] 7.7; 95% confidence interval - 95% CI 3.8 - 15.6; p = 0.000) and age > 50 years (OR 2.4, 95% CI 1.1 - 5.4, p = 0.02) (Table 4).

**Table 4:** Multivariate analysis of factors associated with hospital mortality

	OR	95%CI	p value
Age > 50 years	2.4	1.1 - 5.4	0.02
SAPS II> 35 points	1.5	0.7 - 3.3	ns
EPOC	1.02	0.4 - 2.5	ns
Cardiopathy	1.6	0.7 - 3.9	ns
Neoplasia	3.3	0.4 - 23.9	ns
Tracheostomy	1.6	0.7 - 3.5	ns
Weekend ICU discharge	7.7	3.8 - 15.6	0.000

OR - odds ratio; CI - confidence interval; SAPS II - Simplified Acute Physiology Score II; COPD - chronic obstructive pulmonary disease; ICU - intensive care unit; ns - not significant.

## DISCUSSION

The prognostic impact of the time of admission to and discharge from the ICU has been evaluated in different studies. The factors that have most often been associated with an increase in mortality for off-hours discharges are difficulty accessing diagnostic or therapeutic procedures, greater severity of patients who arrive on weekends and at night, and a lack of 24-hours access to intensive care doctors.^(1-4,14-16)^

When we analyzed our population, we did not find differences in patient according to the day or time of admission. In this context, there were no differences in patient mortality linked to the day or time of admission to the ICU. It should be noted that the analyzed hospital has intensive care doctors available in the ICU 24 hours per day every day of the year and similar access to diagnostic techniques at different times and days of the week. These factors may explain the lack of an effect on mortality by the time of ICU admission at this hospital.

The time at which the patient is discharged had a special prognostic implication in our study. Discharges to the floor during the weekend and mainly on Fridays were associated with higher hospital mortality rates. Several European authors have found similar results linked to off-hours discharges.^(1,7,9,11,17-21)^ An evaluation of the causes associated with this lower survival is beyond the scope of this study, which was not designed for that purpose. Nevertheless, the increase in absenteeism on weekends combined with the treatment changes that result from transfer to the hospital room can explain this increase in mortality.^(22,23)^ When evaluating the mortality rate for the 3 days of the weekend, we observed that the highest mortality occurred on Friday. Discharges on Sunday were associated with better prognoses. Like the results of the study that Laupland conducted in France, this finding raises the hypothesis that patients who are exposed to greater risk associated with reduced human resources or probable inconsistencies in care face a worse prognosis in the hospital.^(12)^

The fact that Fridays are associated with a worse prognosis can be explained by the greater amount of time patients admitted on Fridays are exposed to the weekend effect. It is likely that the high mortality of our patients on the hospital floor was the results of a clinical deterioration that went unnoticed during the transition of care on the weekend.

This problem will be challenging to resolve. Not allowing weekend discharges and prolonging patients' stays does not seem a reasonable solution, nor is increasing the number of beds feasible in the short-term. However, promoting a culture of safety and integrating it into the hospital's strategy can help resolve this conflict. In this scenario, strategies for improving the perception of risks, the safety of patients outside the ICU and the management of clinical deterioration may have a beneficial effect on mortality.^(24-26)^ Early detection, follow-up at discharge, active screening for at-risk patients, and the activation of resources when alarming signs and symptoms appear reduces the risk of inadvertent clinical deterioration.^(27-29)^ Expanding intensive care services outside the unit walls and developing critical care transition programs with the activation of rapid response teams, ICU extension teams, or ICU nursing liaison programs has proven to effectively reduce the weekend effect and increase patient safety.^(30-33)^

This study has limitations that must be considered when interpreting its results. It was conducted in a single center, so it only demonstrates the situation in that center over a specific period, and its results cannot be generalized to other settings. Additionally, the definitions of weekend and off-hour admission were arbitrarily established. Different studies have used different definitions.^(18)^ Finally, our study focused on the ICU setting and evaluated data from patients' stay in the unit; therefore, it lacks detailed data on the aftercare provided in the rooms. Consequently, we can only generate hypotheses regarding the increase in mortality during the weekends.

## CONCLUSION

This study shows that factors in addition to those related to the severity of the critical patient are associated with patient prognosis. Both the structure and the process of care impact the final outcome. In this sense, the time of discharge is independently associated with hospital mortality.
